# Robust and Transparent Silver Oxide Coating Fabricated at Room Temperature Kills *Clostridioides difficile* Spores, MRSA, and *Pseudomonas aeruginosa*

**DOI:** 10.3390/microorganisms12010083

**Published:** 2023-12-31

**Authors:** Mohsen Hosseini, Jinge Huang, Myra D. Williams, Gerardo Alexander Gonzalez, Xiuping Jiang, Joseph O. Falkinham, William A. Ducker

**Affiliations:** 1 Department of Chemical Engineering, Center for Soft Matter and Biological Physics, Virginia Tech, Blacksburg, VA 24061, USA; mohsenhosseini@vt.edu (M.H.); jerry19@vt.edu (G.A.G.); 2 Department of Food, Nutrition, and Packaging Sciences, Clemson University, Clemson, SC 29634, USA; jingeh@g.clemson.edu (J.H.); xiuping@clemson.edu (X.J.); 3 Department of Biological Sciences, Virginia Tech, Blacksburg, VA 24061, USA; mywillia@vt.edu (M.D.W.); jofiii@vt.edu (J.O.F.III)

**Keywords:** transparent, coating, robust, silver, Ag_2_O, antibacterial, antimicrobial, bacteria, spore

## Abstract

Antimicrobial coatings can inhibit the transmission of infectious diseases when they provide a quick kill that is achieved long after the coating application. Here, we describe the fabrication and testing of a glass coating containing Ag_2_O microparticles that was prepared from sodium silicate at room temperature. The half-lives of both methicillin-resistant *Staphylococcus aureus* (MRSA) and *Pseudomonas aeruginosa* on this coating are only 2–4 min. The half-life of *Clostridioides difficile* spores is about 9–12 min, which is extremely short for a spore. Additional tests on MRSA demonstrate that the coating retains its antimicrobial activity after abrasion and that an increased loading of Ag_2_O leads to a shorter half-life. This coating combines the properties of optical transparency, robustness, fast kill, and room temperature preparation that are highly desirable for an antimicrobial coating.

## 1. Introduction

Bacteria play a significant role in causing many healthcare-related diseases and deaths. They are transferred between people via a variety of mechanisms (skin-to-skin contact, droplet-borne, airborne, vector-borne, etc.) [1], but our particular interest here is in bacteria that are transmitted via inanimate surfaces. Bacteria remain viable on solids for an extended period (hours to months) [2]. Human contact with handrails, doorknobs, touchscreens, buttons, etc., is a route to transmission [3,4,5,6], and such contacts occur frequently in hospitals. Our overarching goal is to reduce the number of healthcare-related infections through the use of antimicrobial coatings that could be applied to common touch surfaces, such as hand-railings, or high-touch surfaces in healthcare settings, etc.

Current methods for reducing surface transmission encompass practices such as hand washing, cleaning, and surface disinfection [7]. However, these methods demand fastidious attention and repetition on a timescale that is shorter than the time between users. The use of chemical disinfectants also comes with its own set of health-related risks, such as potential harm to the eyes, skin, and respiratory tract [8]. Additionally, some disinfectants, such as bleach, are environmentally harmful, so continual application is not ideal.

Coatings are used in various applications [9,10,11,12], and an alternative or complementary approach for mitigating infection via surfaces is the development and implementation of antimicrobial coatings. These coatings operate on two timescales. They must provide (1) a fast kill: a kill time faster than the period between users, and (2) an ongoing kill: the fast kill should be effective over weeks, months, or even years in order to save the cost and inconvenience of reapplying the coating.

Antibacterial coatings have been reviewed [7,13,14,15,16], and metal oxide-based coatings containing silver (Ag), zinc (Zn), or copper (Cu) are proven to kill bacteria and viruses such as MRSA [17], *E. coli* [18], influenza A [19], SARS-CoV-2 [20,21], and norovirus [22]. The speed at which antimicrobial coatings kill bacteria is of paramount importance.

In this study, we use silver oxide (Ag_2_O) as the active ingredient of a coating. Ag_2_O has been widely used in the medical industry due to its robust mechanical properties and biocompatibility [23,24,25,26,27] and for antimicrobial materials [17,28,29,30,31,32,33,34]. For example, Tsendzughul et al. [24] fabricated an optically transparent film by sputtering silver oxide on a surface. A significant concern regarding the use of silver oxide is whether it exhibits any cytotoxic effects. Silver oxide has shown no sign of cytotoxicity against L929 fibroblast cells [29] and G292 osteoblastic cells [25]. A study onusing silver and silver oxide as an antimicrobial coating on footwear demonstrated impressive antibacterial properties without cytotoxicity [26]. Silver oxide is also used in urinary catheters to enhance infection resistance and has shown antimicrobial efficacy while remaining non-cytotoxic [27].

Our objective was to design and test a transparent and robust silver oxide antimicrobial coating that is fabricated at room temperature. A transparent coating is a necessity for important applications such as touchscreens and is desirable in many applications because of their aesthetics.

Test organisms for antimicrobial coatings should be those that are both medically significant pathogens and have significant transmission via surfaces. We test our coatings against *Pseudomonas aeruginosa* (*P. aeruginosa*), methicillin-resistant *Staphylococcus aureus* (MRSA), and *Clostridioides difficile* (*C. difficile*). *P. aeruginosa* (Gram-negative) is a significant cause of community and hospital-acquired infections and can be transferred through contaminated objects (fomites) [35]. *P. aeruginosa* spreads to organs that have already been damaged and those with implants [36]. *P. aeruginosa* causes between 10% and 20% of infections in most hospitals [37]. MRSA (Gram-positive) is an antibiotic-resistant strain of *Staphylococcus aureus* that causes pneumonia, sepsis, and skin infections. MRSA is typically found on the skin or nose. It can remain viable on surfaces for as long as months [2,38] and can be transmitted through direct contact or contaminated surfaces [39]. *C. difficile* is an anaerobic, Gram-positive spore-forming bacillus that is primarily found in the intestinal tract of humans and animals [40,41] and can cause diarrhea, colitis, and septicemia, potentially resulting in death [42]; in the US, 500,000 people annually are affected by this bacterium [43]. *C. difficile* is known to persist and spread through inanimate surfaces [44], surviving for up to 5 months on surfaces [44]; therefore, it is a good target for antimicrobial coatings.

Here, we describe a novel, transparent, and highly robust antimicrobial coating. The coating is primarily silicate glass, which is a robust and transparent material. Our design was to use 2 µm particles as a compromise between small particles to achieve a high surface-to-volume ratio while avoiding nanoparticles because of potential toxicological effects due to easier cellular entry [45]. We wanted the silver particles to span the coating, so the coating was less than 2 µm thick by design. Thicker coatings would submerge some particles, and thinner coatings would be less robust. The matrix was prepared by room-temperature spin coating of a sodium silicate solution containing a suspension of Ag_2_O particles. Very good antimicrobial properties were achieved: >3 logs (99.9%) of kills within 40 min for MRSA and *P. aeruginosa,* and 1.84 logs (98.6%) of kills within 60 min against *C. difficile* endospores.

In a previous work [17], we fabricated an optically transparent film by employing a variant of the Stöber process to bind silver oxide to surfaces, followed by a heat treatment at 50 °C for 40 min. Fabrication at 50 °C is a practical disadvantage for coatings applied to existing infrastructure; typical infrastructure, such as a touch screen or railing, cannot be maintained at 50 °C in the field. Here, we describe a novel coating method that can be applied at room temperature, overcoming this limitation. The new coating method also has the following advantages over the previous method: it eliminates a 40-h reaction with ammonia, a category 3 toxin that causes acute hazards to the aquatic environment [46] and, therefore, is less suitable for field application. This is achieved by using a different coating method based on sodium silicate. By eliminating ammonia, we also eliminate a (Lewis base) ligand that binds strongly to metal cations [47]. As a result, in contrast to previous work, we are able to maintain the morphology of the Ag_2_O particles during the fabrication of the coating. This advantage will likely apply to other particles that react or dissolve in ammonia. Longevity and resistance to abrasion are important to increase the period between repeated applications of the coating. Here, we also show that the new coating is highly abrasion-resistant by demonstrating the antimicrobial properties after abrasion. Compared to our previous Ag_2_O coating [17], the new coating is more potent and kills almost 2 logs of MRSA in only 20 min, whereas the previous coating did not produce a measurable kill in this time. The new coating kills almost two logs of *C. difficile* endospores in 60 min. This is particularly notable because, despite its clinical importance, we find no reports of similar or better killing of *C. difficile* by any coating in the literature.

## 2. Materials and Methods

### 2.1. Materials

Silver nitrate (AgNO_3_) 99.9% and ammonia solution certified as ACS Plus were purchased from Fisher Scientific (Waltham, MA, USA). Sodium hydroxide pellets (NaOH, ACS grade), 100% Ethanol (EtOH, ACS grade), nitric acid (ACS grade), and glass slides measuring 25 × 75 × 1 mm were obtained from VWR (Radnor, PA, USA). Sodium silicate solution (catalog model N) was generously provided by PQ Corporation (Malvern, PA, USA). Deionized (DI) water was used from a Milli-Q Reference (MilliporeSigma, Burlington, MA, USA) water purification system. All water used in the preparation of the coatings was purified water from the Milli-Q Reference system.

### 2.2. Ag_2_O Microparticle Synthesis

The synthesis of silver oxide microparticles has been discussed previously [48]. Here, 200 mL of aqueous 0.1 M AgNO_3_ was stirred while 400 mL of aqueous 0.1 M ammonia was introduced dropwise, stirred for an additional 10 min, and then 20 mL of 2 M NaOH solution was slowly added. This introduction of NaOH caused the solution to transition into a deep brown color, signaling the formation of silver oxide precipitates. The resulting suspension was left undisturbed at room temperature overnight, during which time, silver oxide particles gradually sedimented. The supernatant was then decanted, and silver oxide particles were rinsed three times with DI water and then three times with ethanol. Finally, the collected particles were allowed to air dry.

### 2.3. Preparation of Silver Oxide Coatings

Glass slides were cut into 15 × 15 mm samples and subjected to a rinse with DI water, ethanol, 6 M nitric acid, and another 3× DI water. A uniform 75% vol. solution of sodium silicate in water was prepared by vortexing for 30 s and then leaving in an ultrasonic bath for 3 min. The viscosity was 1.08 mPs and pH was 11; Ag_2_O particles are resistant to this pH. This solution was used to create a 13.5% wt. silver oxide in sodium silicate suspension that was homogenized by vortexing for 30 s and ultrasonic waves for >10 min. Glass pieces underwent O_2_ plasma cleaning at 100 W with a pressure of less than 200 torr for 4 min and then were immediately positioned on a spin coater. A 100 µL suspension solution was applied to the surface of the substrate and spin-coated for 30 s at 1200 rpm and with a startup acceleration of 3000 rpm/s. The resulting samples are described as the “Ag_2_O coating” in the remainder of this paper.

### 2.4. Characterization of Microparticles and Coatings

The crystal structure of the synthesized Ag_2_O particles was determined by analyzing the X-ray diffraction (XRD, Bruker D8 Advance diffractometer with a monochromatic Cu Kα X-ray source with a wavelength of 1.5418 Å). The peaks in the range of 2θ = 20–80° were compared to the known structure of Ag_2_O to check consistency with the product being crystalline Ag_2_O. The chemical composition of the few outer nanometers of the surface of the Ag_2_O coating was obtained using a survey spectrum using X-ray photoelectron spectroscopy (XPS, PHI VersaProbe III (Chanhassen, MN, USA) with Al Kα source at 1486.6 eV). The coating morphology was examined using scanning electron microscopy (SEM, JEOL, Japan JSM-IT500). The sample was sputtered with 5 nm of iridium in a no-tilt position to increase the signal-to-noise ratio during the SEM imaging of the nonconductive materials. Optical transmittance measurements were performed using an Agilent model 8453 UV−Vis spectrometer. Air was used as the blank spectrum.

### 2.5. Microbial Strains

We utilized *P. aeruginosa* strain DSM-9644, *C. difficile* (ATCC 43593) endospores, and a strain of methicillin-resistant *Staphylococcus aureus* (MRSA) known as MA43300, which was sourced from Danville Community Hospital in Danville, Virginia.

### 2.6. Growth of Microbial Strains

*P. aeruginosa* and MRSA strains were cultured in 5 mL of Tryptic Soy Broth (TSB) and were grown to the mid-exponential phase at 37 °C with continuous aeration at 60 rpm. After the growth phase, we confirmed the purity and identity of the cells in the cultures by streaking the bacterial cultures onto Tryptic Soy Agar (TSA) from BD (Sparks, MD, USA) and incubating them at 37 °C for 48 h. During this period, we examined the colonies for species-specific characteristics, such as pigmentation and surface texture. Cultured cells were harvested through centrifugation at 5000× *g* for 20 min. Afterward, the supernatant medium was removed, and the cells were resuspended in 5 mL of sterile phosphate-buffered saline (PBS) by vortexing for 60 s. These suspensions were subjected to another round of centrifugation at 5000× *g* for 20 min, and the supernatant wash was discarded. Subsequently, the washed cells were resuspended in 5 mL of sterile PBS by vortexing for an additional 60 s. To determine the density of colony-forming units (CFU) per milliliter in each of the washed suspensions, we plated 0.10 mL of serial dilutions in PBS onto TSA plates.

*C. difficile* (ATCC 43593) was cultured on modified brain heart infusion agar plates containing 5 g/L yeast extract, 1 g/L cysteine, and 1 g/L sodium taurocholate (BHIA/YE/CYS/T), and incubated inside an anaerobic chamber (BactronEZ, Sheldon Manufacturing, OR, U.S.) at 37 °C for 7 days as previously described [49]. Then, all plates were sealed with Parafilm™ (Pechiney, IL, USA) and incubated under ambient conditions for another 7 days. Each agar plate was flooded with 5 mL of 0.01 M phosphate-buffered saline (PBS) with 0.1% (vol/vol) Tween-80, and the colony mass was scraped from the agar plates using sterile cotton swabs. The cell suspension was washed 5 times with ice-cold sterile deionized (DI) water, followed by centrifugation at 7000× *g* for 5 min at 4 °C. Vegetative cells of *C. difficile* were removed by gradient centrifugation in 50% (*w*/*v*) sucrose solution [50], then the endospore suspension was washed three times with sterile ice-cold water. The concentration of endospores was enumerated on BHIA/YE/CYS/T plates, and the purity of prepared endospores was confirmed under a microscope after endospore staining [51]. The stock culture of *C. difficile* endospores at 10^8^ colony-forming units (CFU)/mL was stored at 4 °C for routine tests and at −80 °C for long-term storage.

### 2.7. Measurement of Cell Number and Surface Killing

#### 2.7.1. *P. aeruginosa* and MRSA

The bacterial cell numbers in the PBS suspensions were measured from the CFU per milliliter of the suspension by spreading 0.1 mL of each solution onto TSA plates in triplicate. Survival on the Ag_2_O coating was determined by depositing a 10 μL droplet of bacterial cell suspension onto each of three separate Ag_2_O coated and uncoated samples at each time-point displayed in the Figures. After predefined time periods, each glass coupon was transferred to an individual sterile 50 mL centrifuge tube containing 5 mL of sterile PBS. Subsequently, the tubes were vortexed for 10 s and sonicated for one minute to release bacteria. A volume of 0.1 mL of the suspension was then spread-plated, which represents 1/50 of the surviving colonies; a series dilution was also plated. Colonies were counted 48 h after incubation at 37 °C. To enable the logarithmic transformation in Equation (1), cases where no colonies were observed for the 1/50 dilution were rounded up to one colony. One colony on the plate is the detection limit displayed in the figures.

#### 2.7.2. *C. difficile* Endospores

Prior to testing the antimicrobial properties, samples were rinsed in 75% ethanol for 10 min and air-dried in 100-mm Petri dishes with lids on under a biosafety cabinet at room temperature (20–25 °C). Twenty microliters of *C. difficile* endospore suspension were inoculated onto the center of each sample and spread to within 3 mm of the edge of each carrier by sterile pipette tips. Triplicate inoculated samples were incubated aerobically at room temperature for up to 60 min. After the predefined incubation times, each sample was immediately transferred to a 50-mL conical tube with 20 mL Dey/Engley neutralization broth. All samples were sonicated at 40 kHz for 5 min and vortexed for 30 s, and the surviving *C. difficile* endospores from each sample were enumerated on anaerobic BHIA/YE/T/CYS plates.

### 2.8. Coating Robustness

The United States Environmental Protection Agency (EPA) has published a protocol [52] for assessing the effectiveness of antibacterial coatings. A sponge (Brite (3M, Saint Paul, MN, USA) Non-Scratch Scrub Sponge, model C05068) used for abrasion was autoclaved, then left to completely dry overnight in a laminar flow hood. Subsequently, the sponge was immersed in 20 mL of 1:6 Lysol in DI water solution for ten minutes, and then the partially wet sponge was affixed to a Gardco model D10 V abrasion tester. The abrasion tester translates the sponge parallel to the active surface of the sample under a load of 0.454 kg, with a period of 2.2 s and a displacement of 0.3 m. Each cycle consists of moving the sponge back and forth over the sample eight times, followed by a 30-min waiting period. Ten such cycles were conducted, totaling 80 passes. Owing to evaporation, the nature of the sponge changed, so cycles 6–10 used a fresh sponge that was also wetted with Lysol solution. Finally, the abraded samples were dipped in sterile deionized water for >10 min and then rinsed 3× with sterile deionized water to remove the remaining Lysol solution.

## 3. Results and Discussion

### 3.1. Coated Glass Is Transparent and Contains Exposed Silver Oxide

**Figure 1:**
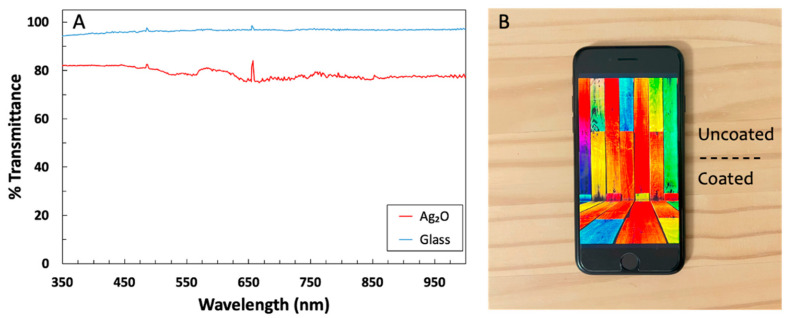
(**A**) Visible light transmission spectrum for a glass slide with the Ag_2_O coating. The background is air in both spectra. (**B**) Photograph of cell phone with an Ag_2_O-coated screen protector on the lower half. Note that various colors from the cell phone screen are transmitted, as suggested by the spectrum in (**A**).

**Figure 2:**
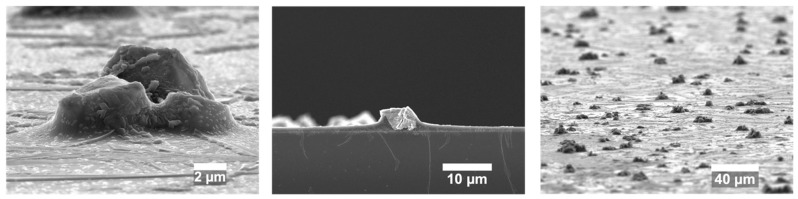
SEM images of Ag_2_O particles embedded in the glass coating at different magnifications.

**Figure 3:**
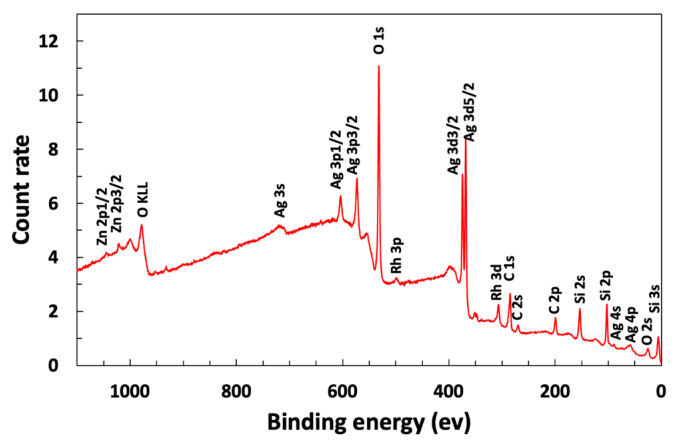
XPS spectrum of the Ag_2_O coating demonstrating that the silver is within a few nanometers of the coating surface.

Glass slides were coated at room temperature with the antimicrobial layer of glass containing Ag_2_O. We synthesized silver oxide microparticles (Figure S1) and confirmed their cubic crystalline structure using XRD. The coated glass was uniformly 80% transparent, as shown by both the transmission spectrum and a photograph of a colored cell phone screen containing a coated glass screen protector (Figure 1). The silver oxide particles protruded beyond the main glass matrix (Figure 2) and, therefore, were suitably positioned for releasing silver ions. SEM images are not sensitive to a thin layer of glass over the particles, so XPS measurements were performed to determine whether the Ag_2_O was exposed. The presence of 6.9 atomic % silver in the XPS spectrum indicated that Ag_2_O was at or within a few nanometers of the coating surface (Figure 3).

### 3.2. The Ag_2_O Coating Has Strong Antimicrobial Activity

**Figure 4:**
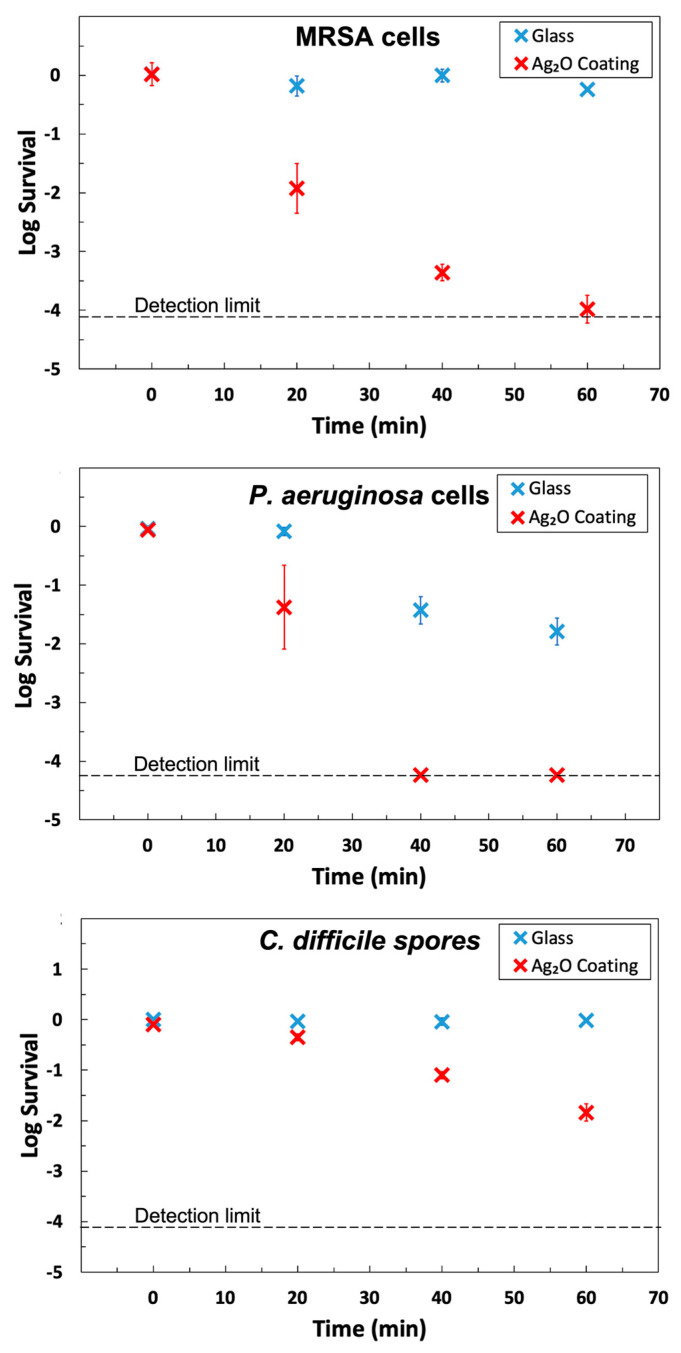
Antimicrobial activity of the Ag_2_O coating towards MRSA and *P. aeruginosa* cells and *C. difficile* endospores. Data is presented for uncoated glass and coated glass at the same exposure time. Survival is defined in Equation (1). Each point represents the average of three independent measurements, and the error bar is the standard deviation of the three points. For MRSA, the 20 min point and 60 min point are the average 5 data points. Two outliers were discarded from the MRSA data due to a large residual from the mean. For both MRSA and *P. aeruginosa*, survival dropped below the detection limit within one hour. There are more survivors for *C. difficile* endospores, but it is a much more difficult organism to kill.

The Ag_2_O coating exhibited strong antimicrobial activity for MRSA, *P. aeruginosa*, and *C. difficile* endospores (Figure 4 and Table 1). The data was plotted as log survival, which is a comparison between the initial titer applied to the solid and the titer recovered from a sample at a designated time:

(1) $$\log\;\mathrm{survival}=\mathrm{mean}\left[\log_{10}\left(\frac{\mathrm{sample}\;\mathrm{titer}}{\mathrm{units}}\right)\right]-\mathrm{mean}\left[\log_{10}\left(\frac{\mathrm{input}\;\mathrm{titer}}{\mathrm{units}}\right)\right]$$

The coating achieved >99.9% killing for both MRSA and *P. aeruginosa* in 40 min, and the half-lives were in the range of 2–4 min (see Table 1). These results meet the standard EPA guideline of 99.9% killing in 60 min and are in agreement with the results for earlier Ag_2_O coatings [17], but the current coatings are superior because they are more robust and are prepared at room temperature.

Our antimicrobial coatings were tested on spore-only suspensions of *C. difficile*. It is much more difficult to kill spores of *C. difficile* than MRSA or *P. aeruginosa* because spores are relatively impermeable, have protective multilayers, and reduced metabolism [53]. The coating showed an outstanding sporicidal response against *C. difficile* by killing 98.55% in one hour. The data for *C. difficile*, shown in Figure 4, exhibits a linear decline of log survival over the entire 60 min time frame, which is characteristic of killing a homogeneous population (cf. data for other organisms in Figure 4) and is consistent with the killing of spores, not the killing of easy-to-kill vegetative cells in a mixture of spores and vegetative cells.

The half-life for *C. difficile* on the coating was about 10 min, which is excellent for spores. Prior publications on both copper and copper-rich alloys (opaque solids) reported that hours were required to kill *C. difficile* [54] or that copper was ineffective [55]. When a germinant was added to the test droplet, the killing of 2.5 logs (99.8% kill) after 3 h was reported. The authors describe this as killing “germinating” cells [55]. In contrast, no germinant was added to the test suspension in the current work.

One can envisage a scenario where *C. difficile* spores in a hospital escape disinfection due to human error and then, even months later, infect another patient. The advantage of a coating is that it can continue to kill *C. difficile* over the long term without human intervention.

### 3.3. Antimicrobial Activity Depends on the Silver Loading

**Figure 5:**
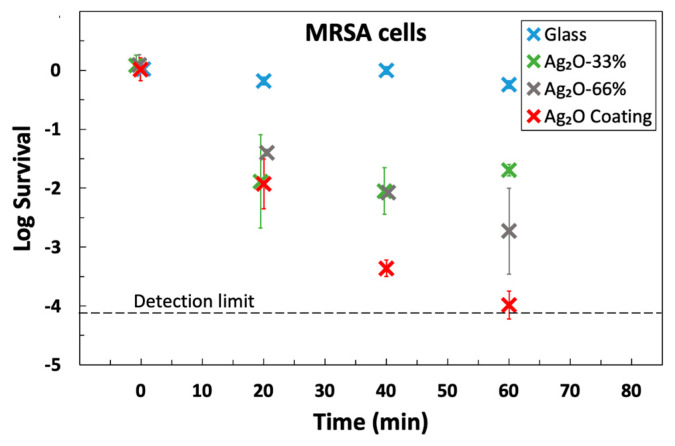
The effect of Ag_2_O loading on antimicrobial activity. The data used for Ag_2_O coating is the same as in Figure 4. The “Ag_2_O Coating” label is for the coating used elsewhere in this manuscript, and Ag_2_O-33% and Ag_2_O-66% indicate coating loadings that have 33% and 66% of that loading, respectively. Data as a function of loading is shown in Figure S3. An increase in loading led to a decrease in the half-life of MRSA.

A control experiment showed that when no silver was added, there was no antimicrobial activity (Figure S2), which is consistent with Ag_2_O being the active ingredient. To determine the dose–response of Ag_2_O in the coating, a series of coatings with equal or lower density than elsewhere in this manuscript was also tested. The three loadings were 0.36 gm^−2^ (33%), 0.73 gm^−2^ (66%), and 1.10 gm^−2^ (100%). The log survival data shows that the rate of killing depended on the loading, which is strong additional support that Ag_2_O is the active ingredient. The results also indicate that the loading is not saturated in this regime, so it is likely that faster killing could be achieved for greater loading. Our hypothesis was that additional Ag_2_O should decrease the half-life. We tested this by fitting all the data used to obtain Figure 5 with a model where log Survival depends on time (min), t, the loading (gm^−2^), l, and an interaction term, tL, with constant coefficients, A, B, C, and D:

(2) log Survival=A−Bt−Cl−Dtl.

The only significant coefficient was for the tl interaction term (*p* = 2 × 10^−8^), demonstrating that increasing the loading decreased the half-life. The fitted half-life in minutes is $t_{1/2}=5.3/l$, where l is the loading in units of gm^−2^.

### 3.4. Antimicrobial Activity Is Retained after Abrasion

**Figure 6:**
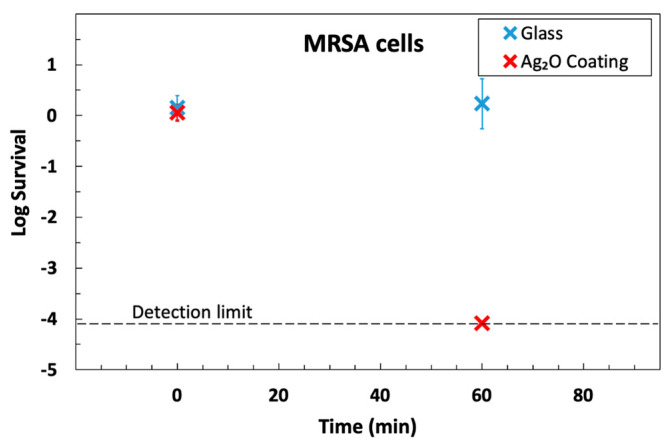
The antimicrobial activity of the Ag_2_O coating is retained after abrasion. The results show a 4-log killing of MRSA on the coating in 60 min.

In practice, antimicrobial coatings are used in environments where they are subject to abrasion. To account for this, the US EPA has published a protocol for testing antimicrobial coatings where they are subject to abrasion [52]. We used the same abrasion cycle and exposure to Lysol disinfectant in a modified version of their protocol and then tested its ability to kill MRSA. The results (Figure 6) show a resistance to abrasion: a 4-log-reduction in 60 min was achieved after abrasion, which is similar to the results prior to abrasion (Figure 4 and Figure 5). The ability of the coating to kill bacteria after light abrasion is not surprising, considering that the coating is mainly composed of glass. We designed a thin coating so that all the particles would protrude. Future work could use a thicker coating such that initially submerged particles could be exposed after abrasion removes the outer layer of glass and, from that time, provide fresh antimicrobial activity for the worn coating.

## 4. Conclusions

Our objective was to fabricate a transparent antimicrobial coating at room temperature, which we achieved by spin-coating a suspension of Ag_2_O microparticles in a sodium silicate solution. The coating was highly effective; it killed >99.9% of *P. aeruginosa*, >99.9% MRSA cells in 40 min, and 98.55% of *C. difficile* spores in 60 min. The results for *C. difficile* spores are particularly notable because they are more difficult to kill. Being primarily glass, the coating is also robust to abrasion and transparent. The combination of transparency, room temperature fabrication, and excellent antimicrobial properties may be useful for combatting the transmission of infectious diseases.

## Figures and Tables

**Table 1:** Statistics summarizing the antimicrobial activity of the Ag_2_O coating.

Organism	Killing, 60 min	Reduction, 60 min	Half-Life (min.) ^1^
MRSA cells	>99.9%	>99.9%	3.3–3.8
*P. aeruginosa* cells	>99.9%	>99.9%	2.6–3.3
*C. difficile* spores	98.55%	98.55%	8.8–11.8

^1^ range indicates a 95% confidence interval. Statistics are for three independent experiments. Equations for killing, reduction, and half-life are in Supplementary Information.

## Data Availability

Data will be provided on request.

## Supplementary Materials

The following supporting information can be downloaded at: https://www.mdpi.com/article/10.3390/microorganisms12010083/s1, Figure S1: Equations used for calculations, further characterization of materials, and data for control coatings. XRD pattern and SEM image of the silver oxide particles. Figure S2: Survival of MRSA cells on a silicate coating with no Ag_2_O. Figure S3: Effect of Ag_2_O loading on antimicrobial activity.
