# Supplementary material for: Robust and Transparent Silver Oxide Coating Fabricated at Room Temperature Kills Clostridioides difficile Spores, MRSA, and Pseudomonas aeruginosa
Source: Microorganisms. 2023 Dec 31;12(1):83. doi: 10.3390/microorganisms12010083 (PMC10818310; doi:10.3390/microorganisms12010083)
Supplement: Supplementary file 1 [file microorganisms-12-00083-s001.zip › microorganisms-2761094-supplementary.pdf]

Supplementary Information

Robust and Transparent Silver Oxide Coating Fabricated at Room Temperature Kills *Clostridioides difficile* Spores, MRSA, and *Pseudomonas aeruginosa*

Mohsen Hosseini,[1] Jinge Huang,[2] Myra D. Williams,[3] Gerardo Alexander Gonzalez,[1] Xiuping Jiang,[2] Joseph O. Falkinham III,[3] William A. Ducker*[1]

*Corresponding Authors' Email: William Ducker, wducker@vt.edu

[1] Dept. of Chemical Engineering and Center for Soft Matter and Biological Physics, Virginia Tech, Blacksburg, Virginia, 24061, USA
[2] Department of Food, Nutrition, and Packaging Sciences Clemson University Clemson South Carolina USA
[3] Department of Biological Sciences, Virginia Tech, Blacksburg, Virginia, 24061, USA

Equations Used for Calculations

Survival and killing are comparisons between the initial titer applied to the solid and the titer recovered from a sample at a time:

$$\text{log survival} = \text{mean}\left[\log_{\mathbf{10}}\left(\frac{\text{sample titer}}{\text{units}}\right)\right] - \text{mean}\left[\log_{\mathbf{10}}\left(\frac{\text{input titer}}{\text{units}}\right)\right] \tag{S1}$$

$$\%\ \text{survival} = \left[1 - 10^{-\text{log survival}}\right] \times 100 \tag{S2}$$

$$\%\ \text{killing} = 1 - \%\ \text{survival} \tag{S3}$$

Reduction compares CFU measurement for two surfaces, an uncoated and a coated surface, at the same time.

$$\text{log Reduction} = \text{mean}\left[\log_{\mathbf{10}}\left(\frac{\text{uncoated surface titer}}{\text{units}}\right)\right] - \text{mean}\left[\log_{\mathbf{10}}\left(\frac{\text{sample titer}}{\text{units}}\right)\right] \tag{S4}$$

$$\%\ \text{Reduction} = \left[1 - 10^{-\text{log Reduction}}\right] \times 100 \tag{S5}$$

Half-life was calculated from a linear fit of the slope of all log (CFU)–time data for a particular coating. The half-life is:

$$t_{1/2} = -\frac{0.30}{\text{slope}} \tag{S6}$$

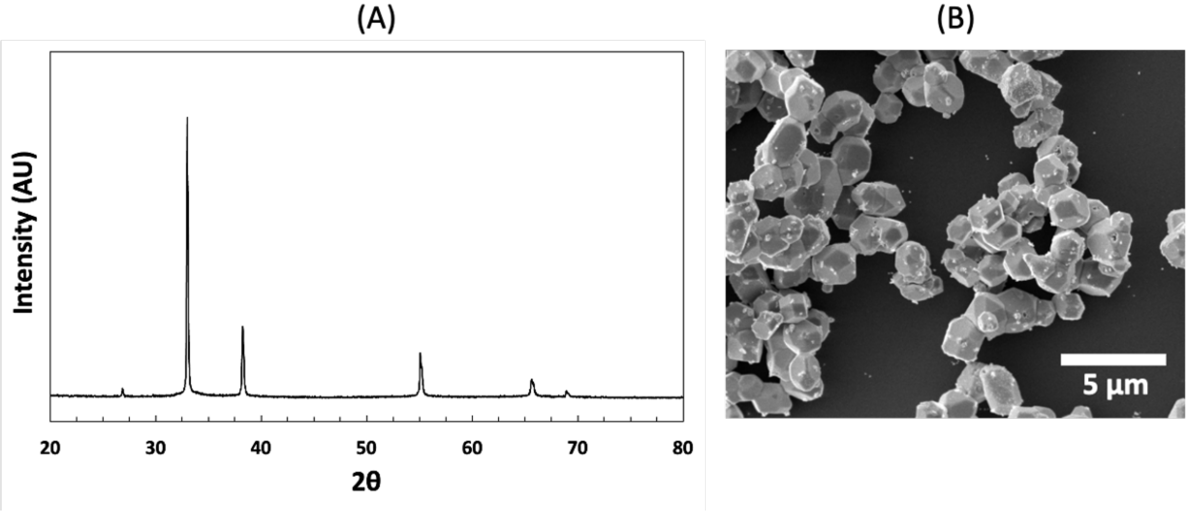

Figure S1. A) XRD pattern of the silver oxide particles demonstrating a cubic crystalline structure. B) SEM image of synthesized Ag_2O microparticles.

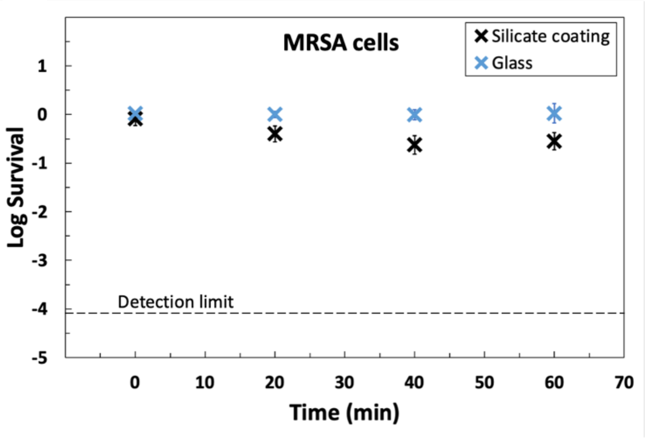

Figure S2. Survival of MRSA cells on a silicate coating with no Ag_2O compared to an uncoated glass slide. The coating was made in the same way as the antimicrobial coatings, but with no Ag_2O. A linear fit to the data for the data for the silicate coating has a slope with a 95% confidence interval in the range -0.0165 to 0.0029, which spans zero and therefore indicates no decline in survival with time.

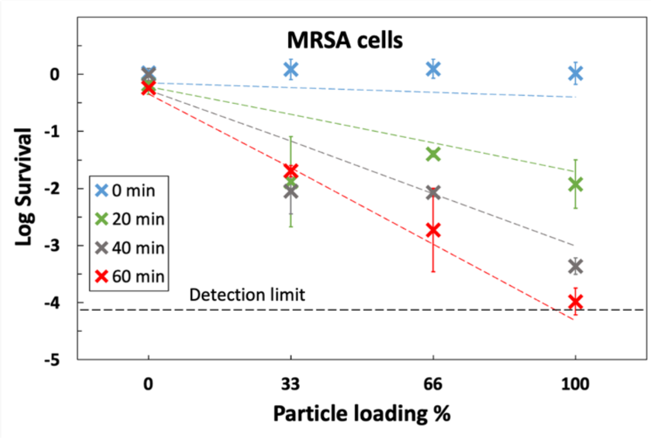

Figure S3. Effect of Ag_2O loading on antimicrobial activity. This is the same data as for Figure 5 but plotted as a function of particle loading. The lines are the plots of Equation 2, each at a fixed time indicated by the figure legend. Overall, an increase in loading led to a decrease in half life.
